# A novel nomogram and risk classification system based on inflammatory and immune indicators for predicting prognosis of pancreatic cancer patients with liver metastases

**DOI:** 10.1002/cam4.6471

**Published:** 2023-08-27

**Authors:** Linjia Peng, Hao Chen

**Affiliations:** ^1^ Department of Integrative Oncology Fudan University Shanghai Cancer Center Shanghai China; ^2^ Department of Oncology, Shanghai Medical College Fudan University Shanghai China

**Keywords:** liver metastases, nomogram, pancreatic cancer, risk stratification system

## Abstract

**Background:**

The study determined to construct a novel predictive nomogram to access the prognosis of pancreatic cancer patients with liver metastases (PCLM).

**Methods:**

Medical records included clinical and laboratory variables were collected. The patients were randomly divided into training and validation cohort. First, in the training cohort, the optimal cutoff value of SII, PNI, NLR, PLR were obtained. Then the survival analysis evaluated the effects of above indices on OS. Next, univariate and multivariate analyses were used to identify the independent factors of OS. Moreover, a nomogram was constructed based on LASSO cox analysis. Additionally, the predictive efficacy of the nomogram was evaluated by ROC curve and calibration curve in the training and validation cohort. Finally, a risk stratification system based on the nomogram was performed.

**Results:**

A total of 472 PCLM patients were enrolled in the study. The optimal cutoff values of SII, PNI, PLR and NLR were 372, 43.6, 285.7143 and 1.48, respectively. By combing SII and PNI, named coSII‐PNI, we divided the patients into three groups. The Kaplan–Meier curves demonstrated above indices were correlated with OS. Univariate and multivariate analyses found the independent prognostic factors of OS. Through LASSO cox analysis, coSII‐PNI, PNI, NLR, CA199, CEA, chemotherapy and gender were used to construct the nomogram. Lastly, the ROC curve and calibration curve demonstrated that the nomogram can predict prognosis of PCLM patients. Significant differences were observed between high and low groups.

**Conclusions:**

The nomogram based on immune, inflammation, nutritional status and other clinical factors can accurately predict OS of PCLM patients.

## Graphical abstract

A novel nomogram and risk classification system for predicting prognosis of pancreatic cancer patients with liver metastases.

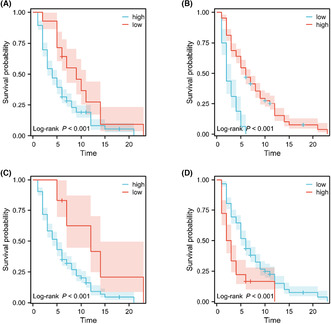

## 1 INTRODUCTION

Pancreatic cancer is a highly heterogeneous malignancy with a 5‐year survival rate of less than 5%, and the majority of patients are often diagnosed with advanced or metastatic pancreatic cancer.^1^, ^2^, ^3^ Pancreatic cancer is prone to metastasis to liver, and the prognosis is extremely poor.^4^, ^5^, ^6^ The progression of pancreatic cancer metastases to liver is a complex process that involves multiple factors and multiple genes. It is undeniable that tremendous progress has been achieved in molecular biology research. Nevertheless, the treatment of PCLM remains controversy. Currently, surgery and chemoradiotherapy are still the major treatments, but the efficacy is not ideal.^7^, ^8^, ^9^, ^10^, ^11^, ^12^ How to prolong the survival time of PCLM patients has been a long‐standing clinical problem, while the prognostic factors of PCLM patients have not been fully understood. Ablation therapy has become a method for the treatment of PCLM, and many studies have proved that it has definite clinical efficacy.^13^, ^14^, ^15^ Therefore, it is urgently needed to assess the prognosis of PCLM patients who underwent ablation.

Increasing evidence suggests that immune status, as well as inflammatory and nutritional status, affects the prognosis of malignant tumors.^16^ It has been shown that inflammation markers, such as systemic immune‐inflammation index (SII), platelet–lymphocyte ratios (PLR), and neutrophil–lymphocyte ratio (NLR), are highly predictive of the outcomes of breast cancer, colorectal cancer, liver cancer, and other cancers.^17^, ^18^, ^19^ The nutritional status of cancer patients also has an impact on the prognosis.^20^ Inflammatory factors and immunity in the body can be reflected more comprehensively by SII, which is a comprehensive index of neutrophil, platelet, and lymphocyte counts. Prognostic nutritional index (PNI) was assessed by serum albumin and lymphocyte counts.^17^ Hence, a novel index composed by SII and PNI was identified. However, there is no study combining immune, inflammation, and nutrition to elaborate the prognosis of PCLM patients.

Therefore, our study explored the prognostic factors for overall survival (OS) in PCLM patients who underwent ablation and established a predictive nomogram model so as to provide clinical decisions and stratified management for PCLM patients.

## 2 METHODS

### 2.1 Patients selection

This study included patients diagnosed with PCLM and hospitalized at Fudan University Shanghai Caner Center between January 2020 and September 2022. The inclusion criteria included: (1) pathologically or cytologically confirmed pancreatic cancer with liver metastases, (2) patient undergoing ablation, and (3) complete clinical data. The exclusion criteria included: (1) patients without liver metastasis and (2) patients without complete clinical information. Four hundred and seventy two patients that met our inclusion and exclusion criteria, with clinicopathological data collected from medical records, were included in our study.

### 2.2 Data collection

According to the characteristics of PCLM, the following clinical information and laboratory data were collected from the results of routing text: age, gender, follow‐up time and status, tumor‐related markers such as carbohydrate antigen 199 (CA199), carbohydrate antigen 125 (CA125), carcinoembryonic antigen (CEA), liver function‐related indicators such as alkaline phosphatase (ALP), alanine transaminase (ALT), aspartate transaminase (AST), and total bilirubin level (TBIL). Laboratory data during routine tests before the first ablation were obtained to ensure uniform baseline data.

### 2.3 Definition of systemic immune‐inflammation index, neutrophil–lymphocyte ratios, platelet–lymphocyte ratios, prognostic nutritional index, and coSII‐PNI

Systemic immune‐inflammation index, prognostic nutritional index, neutrophil/lymphocyte ratio, and platelet/lymphocyte counts ratio were calculated through the following formula: SII = P × N/L; PNI = ALB (g/L) + 5 × L (10^9^/L); NLR = N/L; PLR = P/L, respectively. Then the above‐mentioned indices were divided into groups based on the optimal cutoff value defined by R software. Then coSII‐PNI was defined by combing SII and PNI. High SII and low PNI scored 0 point was defined as group 1, high SII and high PNI or low SII and low PNI scored 1 point was defined as group 2, and low SII and high PNI scored 2 points was defined as group 3.^17^ P, N, and L represent platelet, neutrophil, and lymphocyte, respectively.

### 2.4 Follow‐up

All the included patients had a regular 3‐month follow‐up visit by telephone or outpatient service. Follow‐up was performed until December 2022 or death occurred. OS was defined as time interval between the first ablation and tumor‐related death in December 2022.

### 2.5 Construction and validation of nomogram for predicting prognosis of pancreatic cancer patients with metastatic

To preferably predict the prognosis of PCLM patients, we conducted least absolute shrinkage and selection operator (LASSO) cox analysis in training cohort to screen variables based on the results of univariate and multivariate analyses. Furthermore, predictive performance of the nomogram was evaluated by receiver operating characteristic curve (ROC) and calibration curve.

### 2.6 Risk classification system based on the nomogram

A risk stratification system was developed based on the individual total score calculated from the nomogram. Using the X‐tile software, the optimal cutoff values for the total points of the training cohort were determined. The differences between different subgroups of OS were estimated and compared by Kaplan–Meier method and the log‐rank test.

### 2.7 Statistical analysis

The data were analyzed statistically using R4.0.3, in which median values and ranges were defined as the quantitative variables, while frequency and percentages were defined as the categorical variables. Training and validation cohorts were obtained through the smaple() function in R software. ROC was utilized to determine the best cutoff values of SII, PNI, PLR, and NLR. The differences between groups were accessed by the Kaplan–Meier method and log‐rank test. ROC was drawn based on cox model and LASSO analysis to analyze the predictive value of the model for the prognosis of pancreatic cancer patients with liver metastasis. The area under the curve (AUC) is used to evaluate the predictive performance of the model. The clinical use of nomogram was evaluated by decision curve analysis (DCA). Univariate and multivariate cox analyses were performed to evaluate the effects of different indicators on patient prognosis, while hazard ratio (HR) and 95% confidence interval (CI) were calculated. *p* < 0.05 was considered statistically significant.

## 3 RESULTS

### 3.1 Baseline characteristics of included patients

A total of 472 patients who meet our criterion were included in our study. The first ablation treatment of all the included patients was operated at Fudan University Shanghai Cancer Center. Among them, 140 (29.7%) were female, and 332 (70.3%) were male with a median age of 59 (rang 51.5, 68). The tumor‐related markers and liver function‐related indicators were shown in Table 1.

**TABLE 1:** The clinicopathologic characteristics of pancreatic cancer patients with liver metastases patients.

Characteristic	Levels	Overall
Gender, *n* (%)	Female	140 (29.7%)
	Male	332 (70.3%)
Location, *n* (%)	Body and tail	260 (55.1%)
	Head and neck	212 (44.9%)
CA199 (U/mL), *n* (%)	>37	392 (83.1%)
	≤37	80 (16.9%)
CA125 (U/mL), *n* (%)	>35	296 (62.7%)
	≤35	176 (37.3%)
CEA (ng/mL), *n* (%)	>5.2	272 (57.6%)
	≤5.2	200 (42.4%)
ALP (U/L), *n* (%)	>125	224 (47.5%)
	≤125	248 (52.5%)
ALT (U/L), *n* (%)	>35	116 (24.6%)
	≤35	356 (75.4%)
AST (U/L), *n* (%)	>40	80 (16.9%)
	≤40	392 (83.1%)
TBIL (μmol/L), *n* (%)	>17	48 (10.2%)
	≤17	424 (89.8%)
Age, median (IQR)		59 (51.5, 68)
Distant metastases (except for liver)	Yes	132 (28%)
	No	340 (72%)
Surgery	Yes	144 (38.8%)
	No	328 (61.2%)
Chemotherapy	Yes	452 (95.8%)
	No	20 (4.2%)

Abbreviations: ALP, alkaline phosphatase; ALT, alanine transaminase; AST, aspartate transaminase; CEA, carcinoembryonic antigen; TBIL, total bilirubin level; TBL, total bilirubin level.

### 3.2 Optimal cutoff value of systemic immune‐inflammation index, prognostic nutritional index, platelet–lymphocyte ratios, and neutrophil–lymphocyte ratios

The optimal cutoff values of the above‐mentioned parameters in training cohort were acquired. The optimal cutoff values of SII, PNI, PLR, and NLR were 372, 43.6, 285.7143, and 1.48, respectively.

On the basis of the optimal cutoff value, the patients were divided into different groups: low SII (≤372, *n* = 100); high SII (>372, *n* = 372); low PNI (≤43.6, *n* = 76); and high PNI (>43.6, *n* = 396); low PLR (≤285.7143, *n* = 392); high PLR (>285.7143, *n* = 80); low NLR (≤1.48, *n* = 52); and high NLR (>1.48, *n* = 420).

### 3.3 Relationship between systemic immune‐inflammation index, neutrophil–lymphocyte ratios, PLR, prognostic nutritional index, coSII‐PNI, and overall survival

**FIGURE 1:**
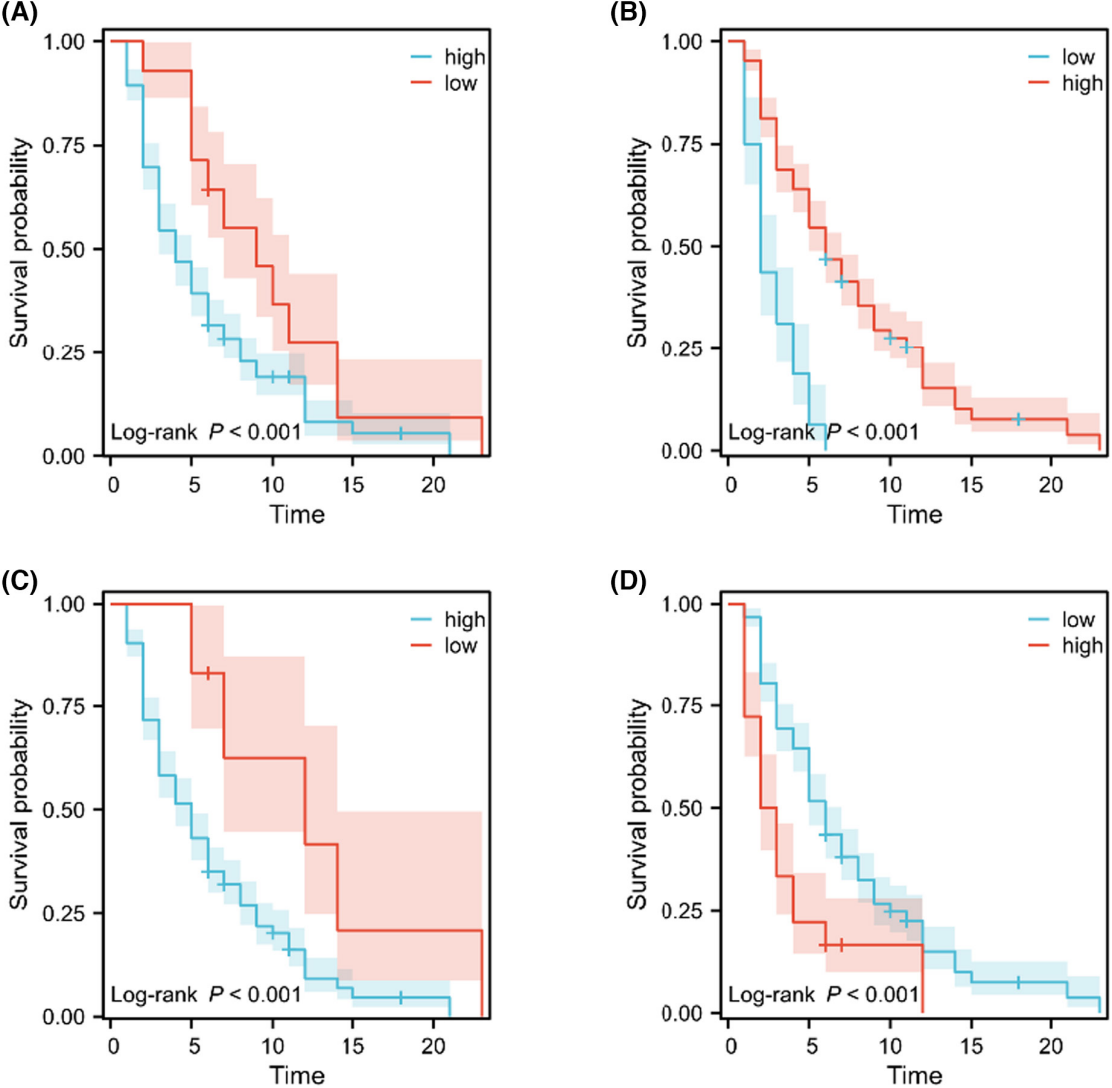
The Kaplan–Meier survival curves for overall survival according to (A) systemic immune‐inflammation index, (B) prognostic nutritional index, (C) neutrophil–lymphocyte ratios, and (D) platelet–lymphocyte ratios in pancreatic cancer patients with liver metastases patients.

**FIGURE 2:**
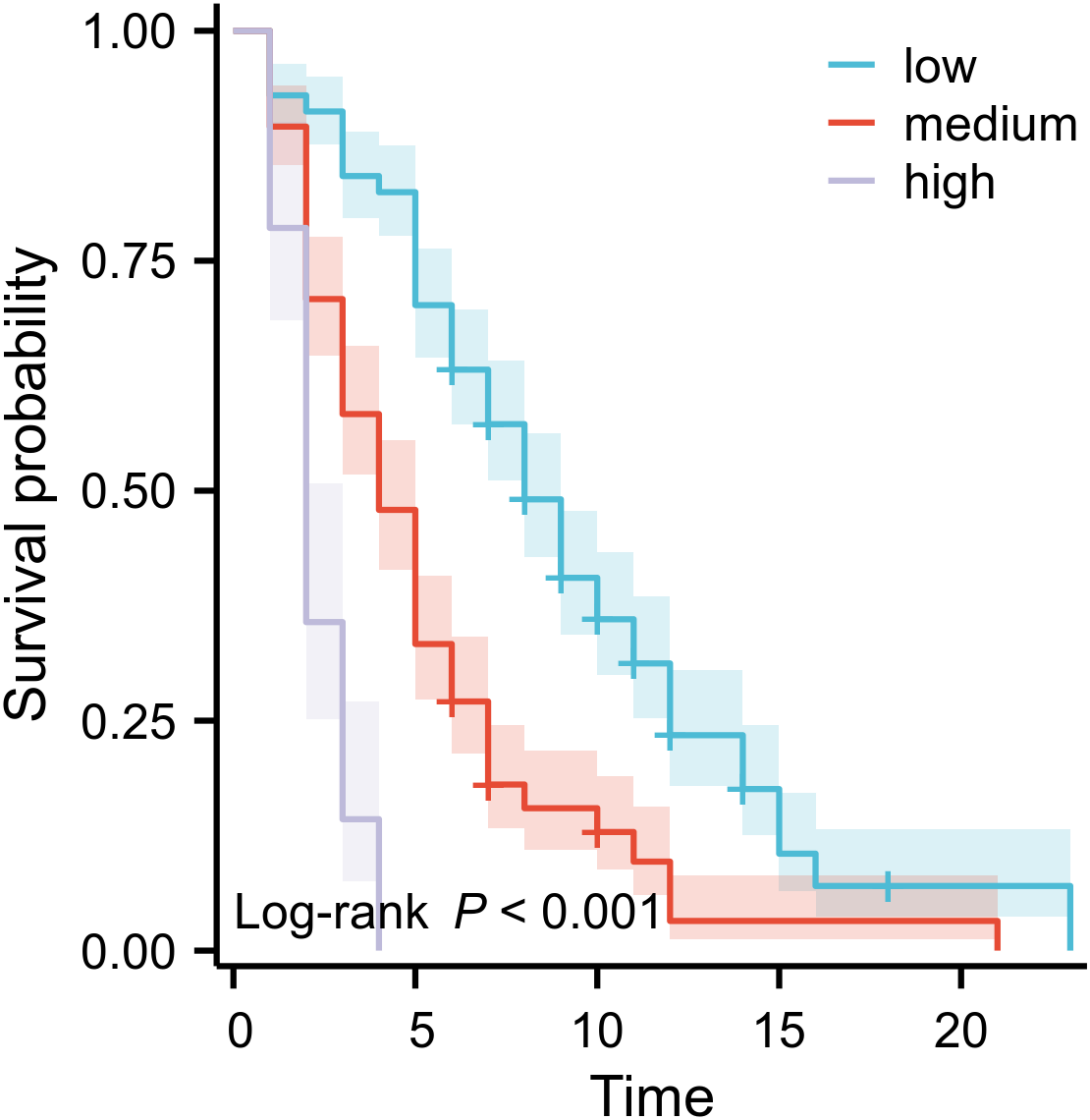
The Kaplan–Meier survival curves for overall survival according to coSII‐PNI in three groups.

In order to explore the relationship of the above‐mentioned indices with OS, the OS of PCLM patients in different level of groups was compared. As shown in Figure 1, patients in a high level of SII (*p* < 0.001; Figure 1A), NLR (*p* < 0.001; Figure 1C), and PLR (*p* < 0.001; Figure 1D) exhibited significantly worse OS, while patients who had a higher level of PNI presented better OS than the patients in the lower level of PNI group (*p* < 0.001; Figure 1B). In addition, referring to coSII‐PNI, patients in different groups demonstrated statistical difference (Figure 2).

### 3.4 Identification of prognostic factors for pancreatic cancer patients with liver metastases patients

**FIGURE 3:**
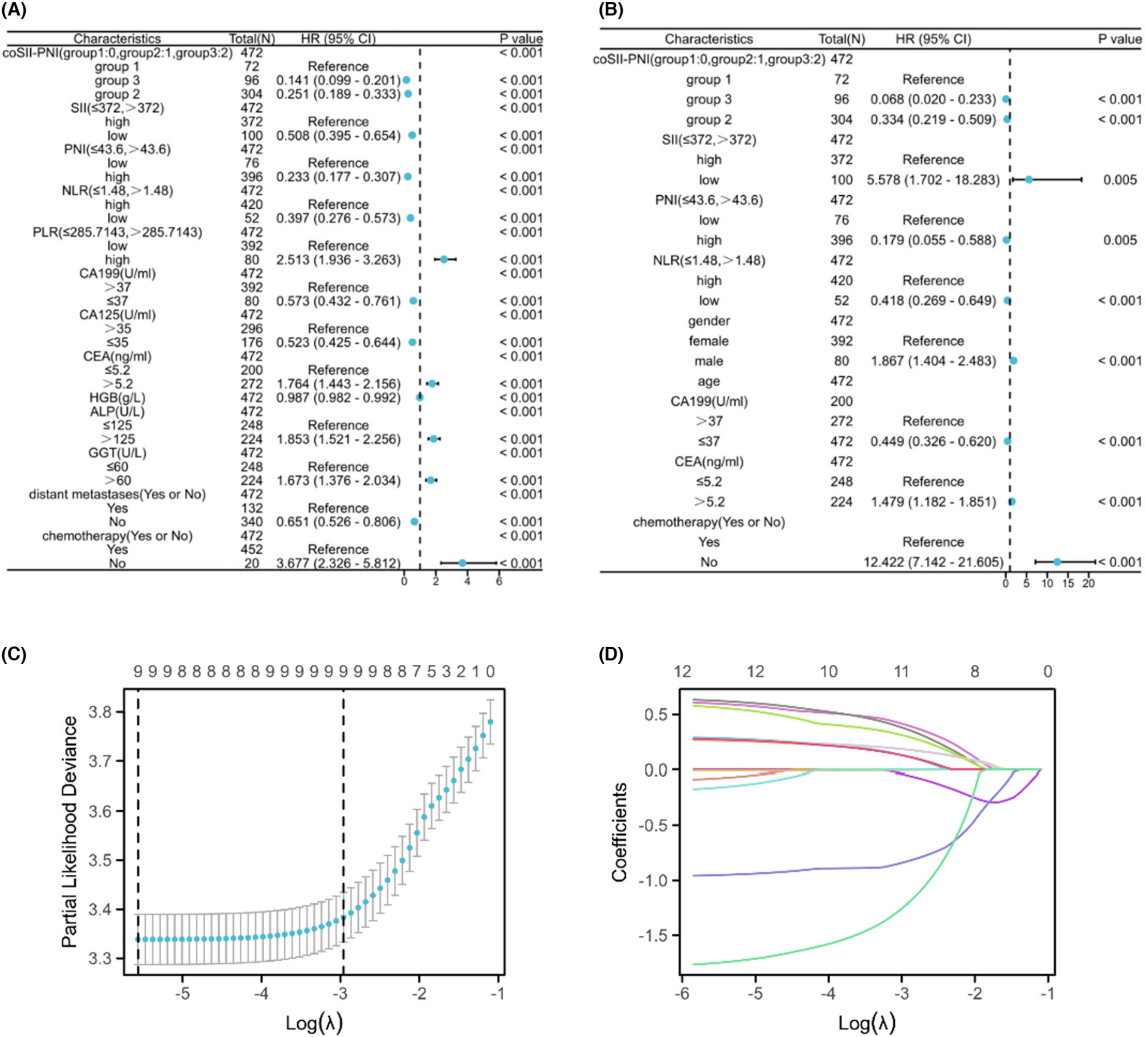
Univariate, multivariate, and least absolute shrinkage and selection operator (Lasso) cox analyses of pancreatic cancer patients with liver metastases patients. (A) Univariate cox analysis; (B) multivariate cox analysis; (C) LASSO coefficient profiles of the features; and (D) binomial deviation and Lasso cox log (λ) curve.

To probe prognostic predictors for PCLM patients, univariate and multivariate cox regression analyses were performed. On the univariate analysis (Figure 3A), coSII‐PNI (*p* < 0.001), SII (*p* < 0.001), PNI (*p* < 0.001), NLR (*p* < 0.001), PLR (*p* < 0.001), CA199 (*p* < 0.001), CA125 (*p* < 0.001), CEA (*p* < 0.001), ALP (*p* < 0.001), chemotherapy (*p* < 0.001), and distant metastases (*p* < 0.001) were significantly associated with OS. Moreover, the results of multivariate analyses (Figure 3B) showed that coSII‐PNI (*p* < 0.001), SII (*p* = 0.005), PNI (*p* = 0.005), NLR (*p* < 0.001), gender (*p* < 0.001), CA199 (*p* < 0.001), CEA (*p* < 0.001), distant metastases (*p* = 0.006), and chemotherapy (*p* < 0.001) were independent prognostic factor with OS. The results were shown in Table 2 and Figure 3A,B.

**TABLE 2:** Univariate and multivariate cox regression analyses of overall survival in pancreatic cancer patients with liver metastases patients.

Characteristics	Total (*N*)	Univariate analysis		Multivariate analysis	
		Hazard ratio (95% CI)	*p* Value	Hazard ratio (95% CI)	*p* Value
CoSII‐PNI (group1:0, group2:1, group3:2)	472		**<0.001**		
Group 1	72	Reference		Reference	
Group 3	96	0.141 (0.099–0.201)	**<0.001**	0.068 (0.020–0.233)	**<0.001**
Group 2	304	0.251 (0.189–0.333)	**<0.001**	0.334 (0.219–0.509)	**<0.001**
SII (≤372, >372)	472		**<0.001**		
High	372	Reference		Reference	
Low	100	0.508 (0.395–0.654)	**<0.001**	5.578 (1.702–18.283)	**0.005**
PNI (≤43.6, >43.6)	472		**<0.001**		
Low	76	Reference		Reference	
High	396	0.233 (0.177–0.307)	**<0.001**	0.179 (0.055–0.588)	**0.005**
NLR (≤1.48, >1.48)	472		**<0.001**		
High	420	Reference		Reference	
Low	52	0.397 (0.276–0.573)	**<0.001**	0.418 (0.269–0.649)	**<0.001**
PLR (≤285.7143, >285.7143)	472		**<0.001**		
Low	392	Reference		Reference	
High	80	2.513 (1.936–3.263)	**<0.001**	1.207 (0.823–1.770)	0.335
Gender	472		0.094		
Female	140	Reference		Reference	
Male	332	1.203 (0.967–1.498)	0.098	1.867 (1.404–2.483)	**<0.001**
Age	472	1.001 (0.990–1.011)	0.890		
Location	472		0.118		
Body and tail	260	Reference			
Head and neck	212	1.170 (0.962–1.423)	0.117		
CA199 (U/mL)	472		**<0.001**		
>37	392	Reference		Reference	
≤37	80	0.573 (0.432–0.761)	**<0.001**	0.449 (0.326–0.620)	**<0.001**
CA125 (U/mL)	472		**<0.001**		
>35	296	Reference		Reference	
≤35	176	0.523 (0.425–0.644)	**<0.001**	0.801 (0.622–1.031)	0.085
CEA (ng/mL)	472		**<0.001**		
≤5.2	200	Reference		Reference	
>5.2	272	1.764 (1.443–2.156)	**<0.001**	1.479 (1.182–1.851)	**<0.001**
ALP (U/L)	472		**<0.001**		
≤125	248	Reference		Reference	
>125	224	1.853 (1.521–2.256)	**<0.001**	0.741 (0.541–1.015)	0.062
ALT (U/L)	472		0.868		
≤35	356	Reference			
>35	116	1.019 (0.812–1.279)	0.868		
AST (U/L)	472		0.140		
≤40	392	Reference			
>40	80	1.231 (0.940–1.612)	0.131		
TBIL (μmol/L)	472		0.394		
≤17	424	Reference			
>17	48	0.870 (0.627–1.206)	0.403		
Distant metastases (Yes or No)	472		**<0.001**		
Yes	132	Reference		Reference	
No	340	0.651 (0.526–0.806)	**<0.001**	0.705 (0.549–0.905)	**0.006**
Surgery (yes or no)	472		0.122		
No	328	Reference			
Yes	144	0.845 (0.680–1.049)	0.126		
Chemotherapy (yes or no)	472		**<0.001**		
Yes	452	Reference		Reference	
No	20	3.677 (2.326–5.812)	**<0.001**	12.422 (7.142–21.605)	**<0.001**

Statistical differences are indicated in bold.

Abbreviations: ALP, alkaline phosphatase; ALT, alanine transaminase; AST, aspartate transaminase; CA199, carbohydrate antigen 199; CEA, carcinoembryonic antigen; NLR, neutrophil–lymphocyte ratios; PLR, platelet–lymphocyte ratios; PNI, prognostic nutritional index; SII, systemic immune‐inflammation index; TBIL, total bilirubin level.

### 3.5 Construction and validation of prognostic nomogram for pancreatic cancer patients with liver metastases patients

**FIGURE 4:**
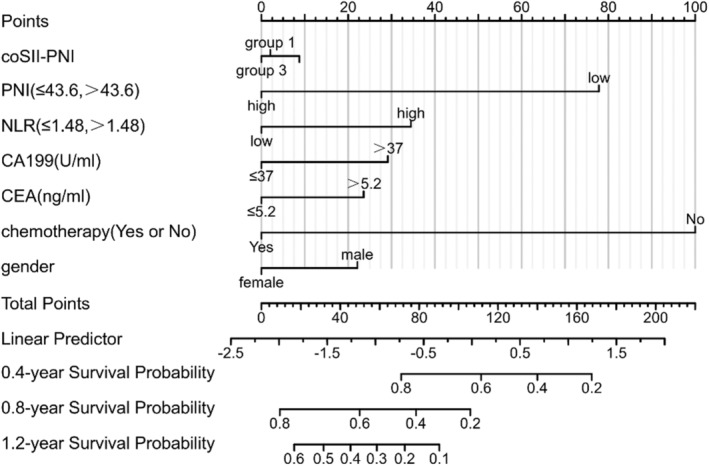
The nomogram model based on coSII‐PNI, prognostic nutritional index (PNI), neutrophil–lymphocyte ratios (NLR), CA199, carcinoembryonic antigen (CEA), gender, and chemotherapy.

The patients were randomly divided into training and validation cohorts in a ratio of 7:3 (Table S1). Based on the results of univariate and multivariate analyses, we included coSII‐PNI, SII, PNI, NLR, CA199, CEA, gender, distant metastases, and chemotherapy in training cohort for further LASSO cox analysis. Through the LASSO analysis, coSII‐PNI, PNI, NLR, CA199, CEA, gender, and chemotherapy were found to have significant difference (Figure 3C,D). Therefore, the above‐mentioned parameters were applied to construct the nomogram for predicting OS in PCLM patients (Figure 4).

**FIGURE 5:**
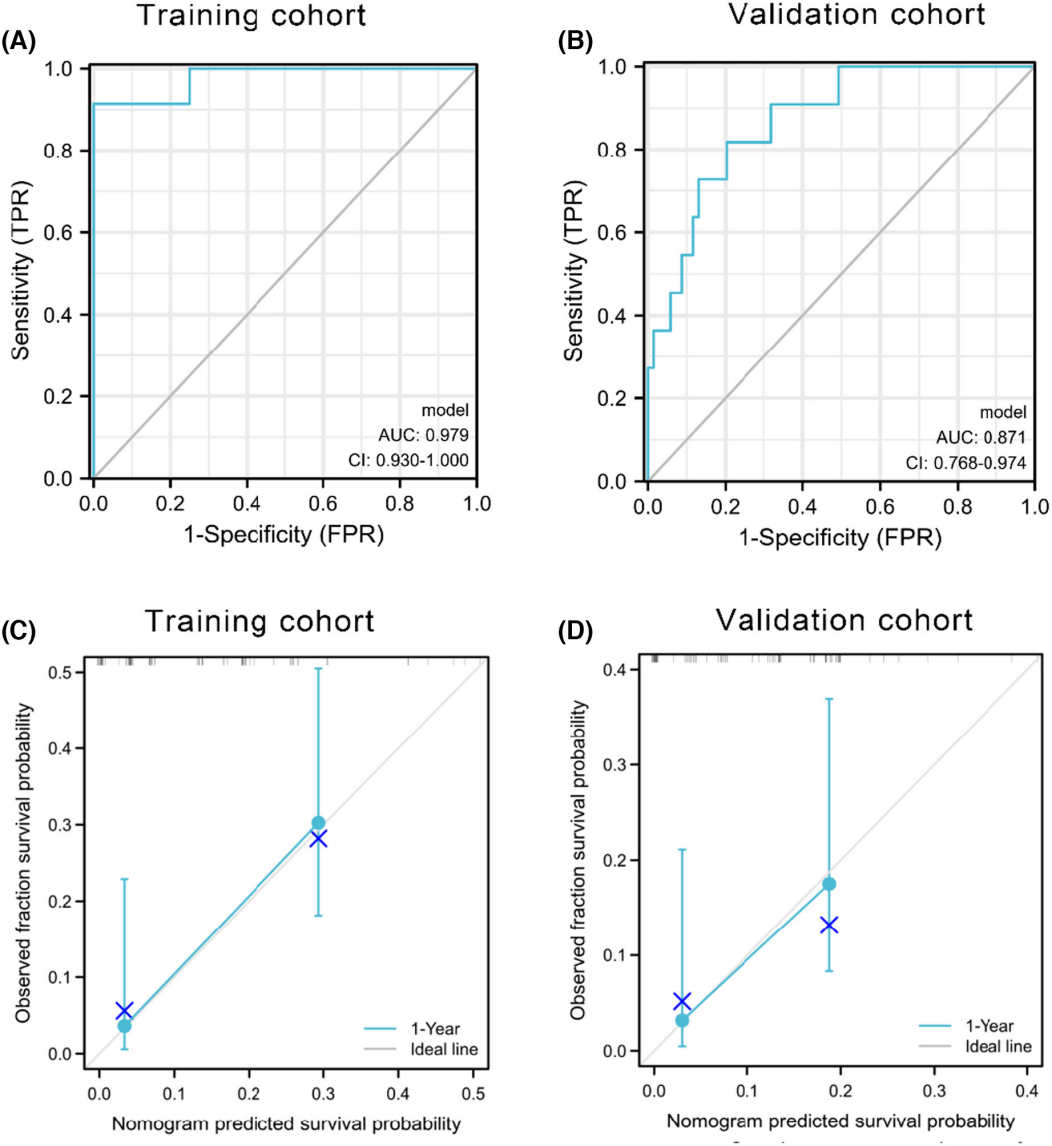
Receiver operating characteristic curve (ROC) and calibration curve. (A) ROC for training cohort; (B) ROC for validation cohort; (C) calibration curve for training cohort; (D) calibration curve for validation cohort.

As shown in the ROC (Figure 5A,B), the AUC values were 0.979 and 0.871 in training and validation cohorts, respectively. Besides, the calibration curve (Figure 5C,D) in training and validation cohorts, respectively, showed the nomogram possessed excellent prognostic value.

### 3.6 Risk stratification based on the prognostic nomogram

**FIGURE 6:**
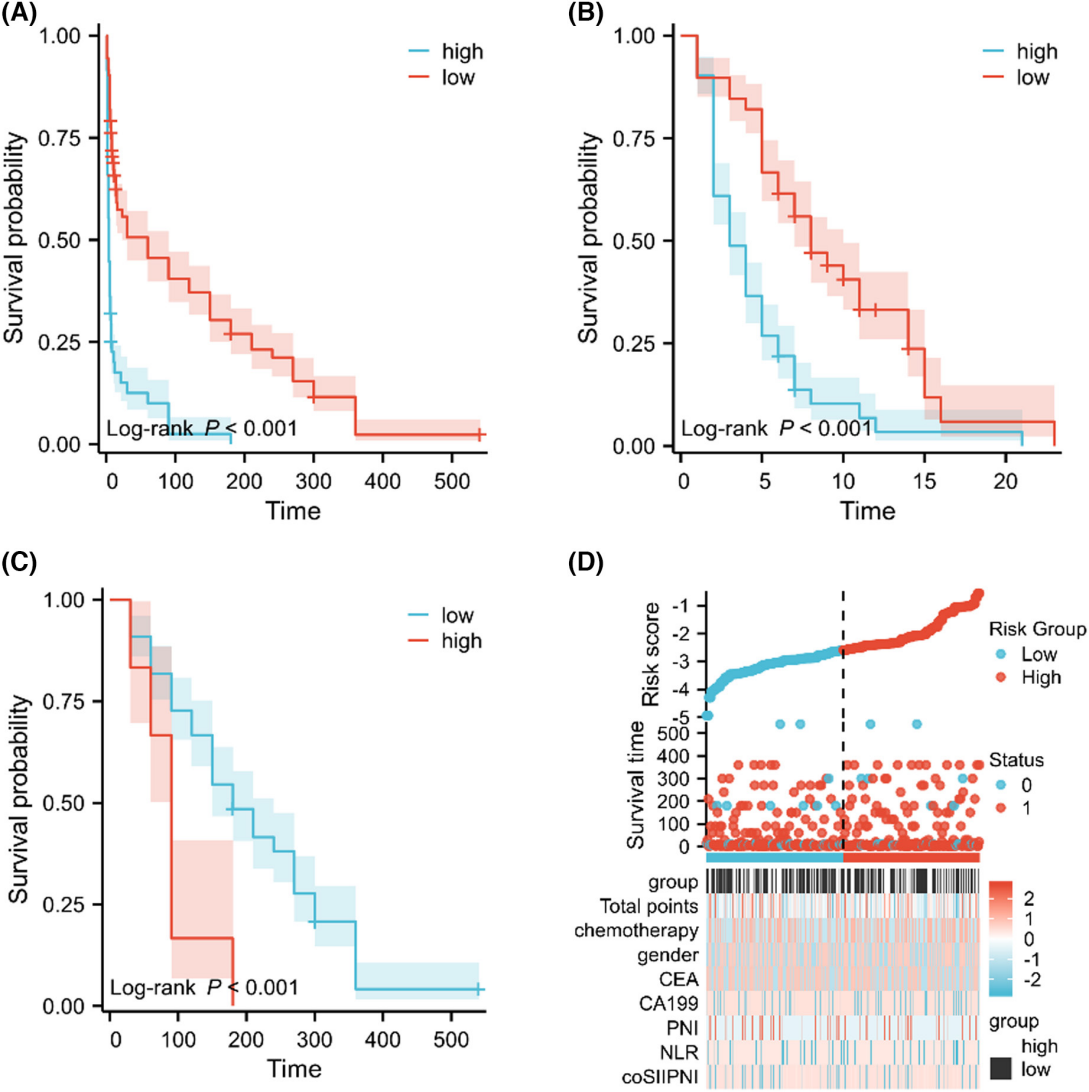
Survival curves of high‐ and low‐risk groups in (A) the whole cohort; (B) training cohort; and (C) validation cohort. (D) The distribution of clinicopathological features in different risk groups for overall survival.

In addition to precisely managing PCLM patients individually, it is essential to classify patients based on their mortality risk. Therefore, a risk stratification system was established to further verify the stability and performance of the nomogram from different dimensions. Specifically, patients' total points were calculated. The optimal cutoff values for the total points were 126 obtained through X‐tile software (Table S2). Then survival analysis showed significant difference in OS between low‐ and high‐risk subgroups (Figure 6A–D).

## 4 DISCUSSION

The onset of pancreatic cancer is insidious, and patients often tend to exist liver metastases, which leads to the limitation of treatment.^5^ Until now, research on whether PCLM patients should be treated surgically has been inconclusive.^8^ Interventional therapy may improve survival, but some of these options are still in their infancy and refinement. Ablation has been an effective approach for the treatment of PCLM.^21^, ^22^ Therefore, it is of great significance to find relevant indicators that can predict the prognosis of patients with pancreatic cancer with liver metastasis to improve the prognosis of patients and reduce the mortality.

The tumor‐associated immune elements in the tumor microenvironment including neutrophils, platelets, and other cells promote tumor progression, invasion, and drug resistance. In addition, these immunoinflammatory cells also contribute to tumor cell extravasation, survival in peripheral blood, and subsequent distant metastasis of tumor cells. Neutrophils can not only enhance the proliferation and spread of cancer cells but also help tumor cells evade surveillance.^23^, ^24^ Lymphocytes play prominent role in inhibiting tumor proliferation and are accompanied by recruiting other immune cells. Platelets carry adenosine trifluridine into the blood circulation, promoting the development of tumors. Platelets are mainly involved in the aggressive behavior of tumors by protecting tumor cells from immune elimination. For example, platelets assist tumor adhesion to evade immune surveillance in pancreatic cancer.^25^, ^26^, ^27^, ^28^ More and more evidence suggests that preoperative status of tumor patients is related to overall prognosis of aggressive tumors, especially nutritional and immune status. High SII, PLR, and NLR mainly indicate an increase in neutrophils and platelets and a decrease in lymphocytes, which means that PCLM patients have a weakened immune response and an enhanced inflammatory response, leading to a poor prognosis.^24^, ^29^, ^30^, ^31^ PNI, as an indicator reflecting the status of nutrition in patients, has been proved to be associated with the prognosis of pancreatic cancer patients.^32^ A new scoring system combined SII with PNI can directly reflect the inflammatory and nutritional status of the body.^19^ The survival analysis demonstrated that high SII, NLR, and PLR were prone to worse OS, whereas high PNI was correlated with better OS. In addition, group 1 which combined with low PNI and high SII represented worse OS.

CA199 and CEA were usually abnormally elevated in PCLM patients, which presents a worse OS.^33^, ^34^, ^35^ PCLM is usually accompanied by abnormal liver function. In our study, most of the patients presented with elevated CA199 and CEA. Clinical examination of liver function indicators can be used to assess the degree of liver function injury and determine whether there is liver metastasis.

According to related reports, nomograms have been applied to predict the survival of pancreatic cancer patients with liver metastases, and most of them applied SEER database data to construct nomogram.^36^, ^37^, ^38^ However, the predictive power of the published models remains unsatisfactory, and they lacked indicators related to immunity and inflammation. In reviewing literature and observing the clinical process, inflammatory, immune, nutritional status, and tumor marker, liver function test is pivotal in PCLM patients, and there are few studies on the application of this method to evaluate and predict the survival of patients. Hence, we developed SII, PLR, NLR, PNI, and coSII‐PNI indices, which combined the immune, inflammatory, and nutritional status and identified three tumor markers and liver function‐related index to explore the prognostic factors correlated with OS of PCLM patients. In our study, on the basis of the univariate and multivariate analyses, coSII‐PNI, SII, PNI, NLR, gender, CA199, CEA, distant metastases, and chemotherapy were found to be the independent prognostic factors of OS.

We identified coSII‐PNI, PNI, NLR, CA199, CEA, gender, and chemotherapy that were significantly correlated with OS. After that, we constructed the nomogram based on the above‐mentioned factors. In addition, ROC and calibration curve showed the nomogram possessed good predictive performance and an agreement between predictions and actual observations. We also develop a mortality risk stratification system to divide patients into high‐ and low‐risk subgroups, enabling clinicians to achieve patient risk stratification management and targeted therapy. The low‐ and high‐risk subgroups demonstrated significant difference in OS. However, the study exists some limitations. Firstly, the sample size of this study is limited, so we will include more patients in the future. Secondly, this is a retrospective study, and there may be a selection bias.

## 5 CONCLUSIONS

In this retrospective study, we have identified prognostic factors related with OS in PCLM patients before ablation. By combining immune, inflammatory, nutritional status, and three tumor markers, liver function‐related index can provide a more comprehensive assessment of PCLM patients' overall physical status. The nomogram emerged superior predictive performance and was able to accurately distinguish low‐ and high‐risk subgroups.

## AUTHOR CONTRIBUTIONS

**Linjia Peng:** Data curation (lead); formal analysis (lead); validation (lead); writing – original draft (lead). **Hao Chen:** Conceptualization (equal); project administration (equal); writing – review and editing (equal).

## FUNDING INFORMATION

This study was supported by the National Natural Science Foundation of China (81973616).

## CONFLICT OF INTEREST STATEMENT

The authors declare that they have no conflicts of interest.

## ETHICS STATEMENT

The study was approved by the ethics committee of Fudan University Shanghai Cancer Center (number 2212267–7). All procedures were in accordance with the ethical standards of the Institutional Research Council and the 1964 Declaration of Helsinki and its later amendments or similar ethical standards. Written informed consent was obtained from patients prior to treatment.

## PATIENT CONSENT STATEMENT

Each participant provided informed consent prior to treatment.

## Supporting information


Table S1

Table S2

## Data Availability

Data sharing is not applicable to this article as no new data were created or analyzed in this study.
