# Supplementary material for: A novel nomogram and risk classification system based on inflammatory and immune indicators for predicting prognosis of pancreatic cancer patients with liver metastases
Source: Cancer Med. 2023 Aug 27;12(18):18622–32. doi: 10.1002/cam4.6471 (PMC10557906; doi:10.1002/cam4.6471)
Supplement: Supplementary file 1 — Table S1 [file CAM4-12-18622-s002.docx]

**Supplementary Table 1**

**Baseline characteristics of training cohort and validation cohort.**

| **Characteristics** | **training cohort** | **validation cohort** | **P value** |
| --- | --- | --- | --- |
| n | 320 | 152 |  |
| gender, n (%) |  |  | 0.136 |
| female | 88 (18.6%) | 52 (11%) |  |
| male | 232 (49.2%) | 100 (21.2%) |  |
| age, median (IQR) | 58 (50.75, 66.25) | 63.5 (53, 70) | < 0.001 |
| location, n (%) |  |  | 0.101 |
| body and tail | 168 (35.6%) | 92 (19.5%) |  |
| head and neck | 152 (32.2%) | 60 (12.7%) |  |
| CA199(U/ml), n (%) |  |  | 0.101 |
| ＞37 | 272 (57.6%) | 120 (25.4%) |  |
| ≤37 | 48 (10.2%) | 32 (6.8%) |  |
| CA125(U/ml), n (%) |  |  | 0.136 |
| ＞35 | 208 (44.1%) | 88 (18.6%) |  |
| ≤35 | 112 (23.7%) | 64 (13.6%) |  |
| CEA(ng/ml), n (%) |  |  | 0.474 |
| ≤5.2 | 132 (28%) | 68 (14.4%) |  |
| ＞5.2 | 188 (39.8%) | 84 (17.8%) |  |
| ALP(U/L), n (%) |  |  | 0.017 |
| ≤125 | 156 (33.1%) | 92 (19.5%) |  |
| ＞125 | 164 (34.7%) | 60 (12.7%) |  |
| ALT(U/L), n (%) |  |  | 0.220 |
| ≤35 | 236 (50%) | 120 (25.4%) |  |
| ＞35 | 84 (17.8%) | 32 (6.8%) |  |
| AST(U/L), n (%) |  |  | 0.643 |
| ≤40 | 264 (55.9%) | 128 (27.1%) |  |
| ＞40 | 56 (11.9%) | 24 (5.1%) |  |
| TBIL(μmol/L), n (%) |  |  | 0.139 |
| ≤17 | 292 (61.9%) | 132 (28%) |  |
| ＞17 | 28 (5.9%) | 20 (4.2%) |  |
| OS（months）, median (IQR) | 5 (2, 8) | 6 (4, 9) | 0.024 |
| status, n (%) |  |  | 0.841 |
| dead | 280 (59.3%) | 132 (28%) |  |
| alive | 40 (8.5%) | 20 (4.2%) |  |
| distant metastases(Yes or No), n (%) |  |  | 0.003 |
| 1 | 76 (16.1%) | 56 (11.9%) |  |
| 0 | 244 (51.7%) | 96 (20.3%) |  |
| surgery(Yes or No), n (%) |  |  | 0.004 |
| 0 | 236 (50%) | 92 (19.5%) |  |
| 1 | 84 (17.8%) | 60 (12.7%) |  |
| chemotherapy(Yes or No), n (%) |  |  | 0.446 |
| 1 | 308 (65.3%) | 144 (30.5%) |  |
| 0 | 12 (2.5%) | 8 (1.7%) |  |
