# Supplementary material for: A novel nomogram and risk classification system based on inflammatory and immune indicators for predicting prognosis of pancreatic cancer patients with liver metastases
Source: Cancer Med. 2023 Aug 27;12(18):18622–32. doi: 10.1002/cam4.6471 (PMC10557906; doi:10.1002/cam4.6471)
Supplement: Supplementary file 2 — Table S2 [file CAM4-12-18622-s001.docx]

**Supplementary Table 2**

**Total points of PCLM patients based on the nomogram.**

| status | Time(months) | Total points | group |
| --- | --- | --- | --- |
| 1 | 5 | 173 | training cohort |
| 1 | 2 | 203 | training cohort |
| 1 | 6 | 110 | training cohort |
| 1 | 7 | 110 | training cohort |
| 1 | 2 | 212 | training cohort |
| 1 | 5 | 120 | training cohort |
| 1 | 12 | 131 | training cohort |
| 1 | 2 | 191 | training cohort |
| 1 | 1 | 212 | training cohort |
| 1 | 9 | 110 | training cohort |
| 1 | 5 | 120 | training cohort |
| 1 | 3 | 147 | training cohort |
| 1 | 21 | 147 | training cohort |
| 0 | 6 | 130 | training cohort |
| 0 | 10 | 126 | training cohort |
| 1 | 2 | 140 | training cohort |
| 1 | 1 | 184 | training cohort |
| 1 | 5 | 126 | training cohort |
| 1 | 4 | 212 | training cohort |
| 1 | 1 | 126 | training cohort |
| 1 | 1 | 124 | training cohort |
| 1 | 7 | 147 | training cohort |
| 0 | 12 | 83 | training cohort |
| 1 | 10 | 119 | training cohort |
| 1 | 5 | 182 | training cohort |
| 1 | 5 | 119 | training cohort |
| 1 | 7 | 138 | training cohort |
| 1 | 4 | 119 | training cohort |
| 0 | 9 | 90 | training cohort |
| 0 | 6 | 46 | training cohort |
| 1 | 4 | 147 | training cohort |
| 1 | 5 | 173 | training cohort |
| 1 | 2 | 203 | training cohort |
| 1 | 6 | 110 | training cohort |
| 1 | 7 | 110 | training cohort |
| 1 | 2 | 212 | training cohort |
| 1 | 5 | 120 | training cohort |
| 1 | 3 | 126 | training cohort |
| 1 | 1 | 117 | training cohort |
| 1 | 15 | 83 | training cohort |
| 1 | 2 | 201 | training cohort |
| 1 | 8 | 73 | training cohort |
| 1 | 11 | 140 | training cohort |
| 1 | 5 | 67 | training cohort |
| 1 | 6 | 147 | training cohort |
| 0 | 8 | 89 | training cohort |
| 1 | 16 | 104 | training cohort |
| 1 | 3 | 140 | training cohort |
| 0 | 14 | 82 | training cohort |
| 1 | 7 | 147 | training cohort |
| 1 | 1 | 126 | training cohort |
| 1 | 1 | 124 | training cohort |
| 1 | 7 | 147 | training cohort |
| 0 | 12 | 83 | training cohort |
| 1 | 10 | 119 | training cohort |
| 1 | 5 | 182 | training cohort |
| 1 | 5 | 119 | training cohort |
| 1 | 7 | 138 | training cohort |
| 1 | 4 | 119 | training cohort |
| 0 | 9 | 90 | training cohort |
| 1 | 4 | 147 | training cohort |
| 1 | 5 | 153 | training cohort |
| 1 | 2 | 147 | training cohort |
| 1 | 7 | 74 | training cohort |
| 1 | 12 | 131 | training cohort |
| 1 | 2 | 191 | training cohort |
| 1 | 1 | 212 | training cohort |
| 1 | 9 | 110 | training cohort |
| 1 | 5 | 120 | training cohort |
| 1 | 3 | 147 | training cohort |
| 1 | 21 | 147 | training cohort |
| 1 | 11 | 117 | training cohort |
| 1 | 6 | 138 | training cohort |
| 1 | 2 | 147 | training cohort |
| 1 | 11 | 110 | training cohort |
| 1 | 8 | 90 | training cohort |
| 1 | 1 | 126 | training cohort |
| 1 | 1 | 117 | training cohort |
| 1 | 15 | 83 | training cohort |
| 1 | 2 | 201 | training cohort |
| 1 | 8 | 73 | training cohort |
| 1 | 11 | 140 | training cohort |
| 1 | 5 | 67 | training cohort |
| 1 | 6 | 147 | training cohort |
| 0 | 8 | 89 | training cohort |
| 1 | 7 | 74 | training cohort |
| 1 | 12 | 131 | training cohort |
| 1 | 2 | 191 | training cohort |
| 1 | 1 | 212 | training cohort |
| 1 | 9 | 110 | training cohort |
| 1 | 5 | 120 | training cohort |
| 1 | 3 | 147 | training cohort |
| 1 | 21 | 147 | training cohort |
| 0 | 6 | 130 | training cohort |
| 0 | 10 | 126 | training cohort |
| 1 | 2 | 140 | training cohort |
| 1 | 1 | 184 | training cohort |
| 1 | 5 | 126 | training cohort |
| 1 | 4 | 212 | training cohort |
| 1 | 2 | 138 | training cohort |
| 1 | 2 | 205 | training cohort |
| 1 | 15 | 83 | training cohort |
| 1 | 2 | 201 | training cohort |
| 1 | 8 | 73 | training cohort |
| 1 | 11 | 140 | training cohort |
| 1 | 5 | 67 | training cohort |
| 1 | 6 | 147 | training cohort |
| 0 | 8 | 89 | training cohort |
| 1 | 16 | 104 | training cohort |
| 1 | 3 | 140 | training cohort |
| 0 | 14 | 82 | training cohort |
| 1 | 7 | 147 | training cohort |
| 1 | 1 | 126 | training cohort |
| 1 | 1 | 124 | training cohort |
| 1 | 7 | 147 | training cohort |
| 0 | 12 | 83 | training cohort |
| 1 | 10 | 119 | training cohort |
| 1 | 5 | 182 | training cohort |
| 1 | 5 | 119 | training cohort |
| 1 | 7 | 138 | training cohort |
| 1 | 4 | 119 | training cohort |
| 0 | 9 | 90 | training cohort |
| 0 | 6 | 46 | training cohort |
| 1 | 4 | 147 | training cohort |
| 0 | 12 | 83 | training cohort |
| 1 | 10 | 119 | training cohort |
| 1 | 5 | 182 | training cohort |
| 1 | 5 | 119 | training cohort |
| 1 | 7 | 138 | training cohort |
| 1 | 4 | 119 | training cohort |
| 0 | 9 | 90 | training cohort |
| 0 | 6 | 46 | training cohort |
| 1 | 4 | 147 | training cohort |
| 1 | 5 | 173 | training cohort |
| 1 | 2 | 203 | training cohort |
| 1 | 6 | 110 | training cohort |
| 1 | 7 | 110 | training cohort |
| 0 | 6 | 126 | training cohort |
| 1 | 3 | 130 | training cohort |
| 1 | 8 | 147 | training cohort |
| 0 | 7 | 140 | training cohort |
| 1 | 6 | 117 | training cohort |
| 1 | 3 | 110 | training cohort |
| 1 | 4 | 191 | training cohort |
| 1 | 1 | 194 | training cohort |
| 1 | 8 | 119 | training cohort |
| 1 | 3 | 164 | training cohort |
| 1 | 15 | 117 | training cohort |
| 0 | 6 | 126 | training cohort |
| 1 | 3 | 130 | training cohort |
| 1 | 8 | 147 | training cohort |
| 0 | 7 | 140 | training cohort |
| 1 | 6 | 117 | training cohort |
| 1 | 3 | 110 | training cohort |
| 1 | 4 | 191 | training cohort |
| 1 | 1 | 194 | training cohort |
| 1 | 8 | 119 | training cohort |
| 1 | 3 | 164 | training cohort |
| 1 | 7 | 74 | training cohort |
| 1 | 12 | 131 | training cohort |
| 1 | 2 | 191 | training cohort |
| 1 | 1 | 212 | training cohort |
| 1 | 9 | 110 | training cohort |
| 1 | 5 | 120 | training cohort |
| 1 | 3 | 147 | training cohort |
| 1 | 21 | 147 | training cohort |
| 0 | 6 | 130 | training cohort |
| 0 | 10 | 126 | training cohort |
| 1 | 2 | 140 | training cohort |
| 1 | 1 | 184 | training cohort |
| 1 | 5 | 126 | training cohort |
| 1 | 4 | 212 | training cohort |
| 1 | 2 | 138 | training cohort |
| 1 | 2 | 205 | training cohort |
| 1 | 23 | 73 | training cohort |
| 1 | 5 | 126 | training cohort |
| 1 | 3 | 128 | training cohort |
| 0 | 11 | 104 | training cohort |
| 1 | 5 | 147 | training cohort |
| 1 | 4 | 147 | training cohort |
| 1 | 2 | 147 | training cohort |
| 1 | 11 | 117 | training cohort |
| 1 | 6 | 138 | training cohort |
| 1 | 2 | 147 | training cohort |
| 1 | 11 | 110 | training cohort |
| 1 | 8 | 90 | training cohort |
| 1 | 1 | 126 | training cohort |
| 1 | 1 | 117 | training cohort |
| 1 | 15 | 83 | training cohort |
| 1 | 2 | 201 | training cohort |
| 1 | 8 | 73 | training cohort |
| 1 | 11 | 140 | training cohort |
| 1 | 5 | 67 | training cohort |
| 1 | 6 | 147 | training cohort |
| 1 | 3 | 126 | training cohort |
| 1 | 2 | 140 | training cohort |
| 1 | 14 | 110 | training cohort |
| 1 | 4 | 147 | training cohort |
| 1 | 5 | 153 | training cohort |
| 1 | 2 | 147 | training cohort |
| 1 | 7 | 74 | training cohort |

| 0 | 7 | 140 | training cohort |
| --- | --- | --- | --- |
| 1 | 6 | 117 | training cohort |
| 1 | 3 | 110 | training cohort |
| 1 | 4 | 191 | training cohort |
| 1 | 1 | 194 | training cohort |
| 1 | 8 | 119 | training cohort |
| 1 | 3 | 164 | training cohort |
| 1 | 1 | 149 | training cohort |
| 1 | 14 | 73 | training cohort |
| 1 | 2 | 147 | training cohort |
| 0 | 7 | 121 | training cohort |

| 0 | 8 | 89 | training cohort |
| --- | --- | --- | --- |
| 1 | 16 | 104 | training cohort |
| 1 | 3 | 140 | training cohort |
| 0 | 14 | 82 | training cohort |
| 1 | 7 | 147 | training cohort |

| 1 | 2 | 140 | training cohort |
| --- | --- | --- | --- |
| 1 | 14 | 110 | training cohort |
| 1 | 4 | 147 | training cohort |
| 1 | 5 | 153 | training cohort |
| 1 | 2 | 147 | training cohort |

| 1 | 1 | 149 | training cohort |
| --- | --- | --- | --- |
| 1 | 14 | 73 | training cohort |
| 1 | 2 | 147 | training cohort |
| 0 | 7 | 121 | training cohort |
| 1 | 4 | 147 | training cohort |
| 1 | 2 | 147 | training cohort |
| 1 | 11 | 117 | training cohort |
| 1 | 6 | 138 | training cohort |
| 1 | 2 | 147 | training cohort |
| 1 | 11 | 110 | training cohort |
| 1 | 8 | 90 | training cohort |
| 1 | 1 | 126 | training cohort |

| 0 | 6 | 46 | training cohort |
| --- | --- | --- | --- |
| 1 | 4 | 147 | training cohort |
| 1 | 5 | 173 | training cohort |
| 1 | 2 | 203 | training cohort |
| 1 | 6 | 110 | training cohort |
| 1 | 7 | 110 | training cohort |
| 1 | 2 | 212 | training cohort |
| 1 | 5 | 120 | training cohort |
| 1 | 3 | 126 | training cohort |
| 1 | 2 | 140 | training cohort |
| 1 | 14 | 110 | training cohort |

| 1 | 1 | 149 | training cohort |
| --- | --- | --- | --- |
| 1 | 14 | 73 | training cohort |
| 1 | 2 | 147 | training cohort |
| 0 | 7 | 121 | training cohort |
| 1 | 4 | 147 | training cohort |
| 1 | 2 | 147 | training cohort |

| 1 | 16 | 104 | training cohort |
| --- | --- | --- | --- |
| 1 | 3 | 140 | training cohort |
| 0 | 14 | 82 | training cohort |
| 1 | 7 | 147 | training cohort |
| 1 | 1 | 126 | training cohort |
| 1 | 1 | 124 | training cohort |
| 1 | 7 | 147 | training cohort |

| 1 | 23 | 73 | training cohort |
| --- | --- | --- | --- |
| 1 | 5 | 126 | training cohort |
| 1 | 3 | 128 | training cohort |
| 0 | 11 | 104 | training cohort |
| 1 | 5 | 147 | training cohort |
| 1 | 15 | 117 | training cohort |
| 0 | 6 | 126 | training cohort |
| 1 | 3 | 130 | training cohort |
| 1 | 8 | 147 | training cohort |
| 0 | 7 | 140 | training cohort |
| 1 | 6 | 117 | training cohort |
| 1 | 3 | 110 | training cohort |
| 1 | 4 | 191 | training cohort |
| 1 | 1 | 194 | training cohort |
| 1 | 8 | 119 | training cohort |
| 1 | 3 | 164 | training cohort |
| 1 | 1 | 149 | training cohort |

| 1 | 2 | 138 | training cohort |
| --- | --- | --- | --- |
| 1 | 2 | 205 | training cohort |
| 1 | 23 | 73 | training cohort |
| 1 | 5 | 126 | training cohort |
| 1 | 3 | 128 | training cohort |
| 0 | 11 | 104 | training cohort |
| 1 | 5 | 147 | training cohort |
| 1 | 15 | 117 | training cohort |
| 0 | 6 | 126 | training cohort |
| 1 | 3 | 130 | training cohort |
| 1 | 8 | 147 | training cohort |

| 0 | 6 | 130 | training cohort |
| --- | --- | --- | --- |
| 0 | 10 | 126 | training cohort |
| 1 | 2 | 140 | training cohort |
| 1 | 1 | 184 | training cohort |
| 1 | 5 | 126 | training cohort |
| 1 | 4 | 212 | training cohort |
| 1 | 2 | 138 | training cohort |
| 1 | 2 | 205 | training cohort |
| 1 | 23 | 73 | training cohort |
| 1 | 5 | 126 | training cohort |
| 1 | 3 | 128 | training cohort |
| 0 | 11 | 104 | training cohort |
| 1 | 5 | 147 | training cohort |
| 1 | 15 | 117 | training cohort |

| 1 | 2 | 212 | training cohort |
| --- | --- | --- | --- |
| 1 | 5 | 120 | training cohort |
| 1 | 3 | 126 | training cohort |
| 1 | 2 | 140 | training cohort |
| 1 | 14 | 110 | training cohort |
| 1 | 4 | 147 | training cohort |
| 1 | 5 | 153 | training cohort |
| 1 | 2 | 147 | training cohort |

| 1 | 14 | 73 | training cohort |
| --- | --- | --- | --- |
| 1 | 2 | 147 | training cohort |
| 0 | 7 | 121 | training cohort |
| 1 | 4 | 147 | training cohort |
| 1 | 2 | 147 | training cohort |
| 1 | 11 | 117 | training cohort |
| 1 | 6 | 138 | training cohort |
| 1 | 2 | 147 | training cohort |
| 1 | 11 | 110 | training cohort |
| 1 | 8 | 90 | training cohort |
| 1 | 1 | 126 | training cohort |
| 1 | 1 | 117 | training cohort |

| 1 | 30 | 37 | validation cohort |
| --- | --- | --- | --- |
| 1 | 270 | 51 | validation cohort |
| 1 | 150 | 58 | validation cohort |
| 1 | 150 | 39 | validation cohort |
| 1 | 210 | 46 | validation cohort |
| 1 | 120 | 46 | validation cohort |
| 1 | 300 | 46 | validation cohort |
| 1 | 360 | 67 | validation cohort |
| 1 | 90 | 166 | validation cohort |
| 1 | 90 | 166 | validation cohort |
| 0 | 180 | 31 | validation cohort |
| 1 | 270 | 31 | validation cohort |
| 1 | 30 | 78 | validation cohort |
| 1 | 210 | 39 | validation cohort |
| 1 | 150 | 60 | validation cohort |
| 1 | 60 | 67 | validation cohort |
| 0 | 180 | 33 | validation cohort |

| 1 | 120 | 67 | validation cohort |
| --- | --- | --- | --- |
| 1 | 30 | 150 | validation cohort |
| 1 | 90 | 67 | validation cohort |
| 1 | 30 | 30 | validation cohort |
| 1 | 60 | 166 | validation cohort |
| 0 | 540 | 37 | validation cohort |
| 1 | 150 | 37 | validation cohort |
| 1 | 60 | 60 | validation cohort |
| 1 | 30 | 37 | validation cohort |
| 1 | 270 | 51 | validation cohort |
| 1 | 150 | 58 | validation cohort |
| 1 | 150 | 39 | validation cohort |
| 1 | 210 | 46 | validation cohort |
| 1 | 120 | 46 | validation cohort |
| 1 | 300 | 46 | validation cohort |

| 1 | 360 | 66 | validation cohort |
| --- | --- | --- | --- |
| 1 | 90 | 166 | validation cohort |
| 1 | 90 | 46 | validation cohort |
| 1 | 270 | 30 | validation cohort |
| 1 | 180 | 149 | validation cohort |
| 0 | 300 | 40 | validation cohort |
| 1 | 300 | 31 | validation cohort |
| 1 | 180 | 39 | validation cohort |
| 1 | 360 | 60 | validation cohort |
| 1 | 240 | 12 | validation cohort |
| 1 | 120 | 67 | validation cohort |
| 1 | 30 | 150 | validation cohort |
| 1 | 90 | 67 | validation cohort |
| 1 | 30 | 30 | validation cohort |
| 1 | 60 | 166 | validation cohort |
| 0 | 540 | 37 | validation cohort |

| 0 | 180 | 33 | validation cohort |
| --- | --- | --- | --- |
| 1 | 90 | 40 | validation cohort |
| 1 | 60 | 37 | validation cohort |
| 1 | 180 | 30 | validation cohort |
| 1 | 360 | 30 | validation cohort |
| 1 | 360 | 66 | validation cohort |
| 1 | 90 | 166 | validation cohort |
| 1 | 90 | 46 | validation cohort |
| 1 | 270 | 30 | validation cohort |
| 1 | 180 | 149 | validation cohort |
| 0 | 300 | 40 | validation cohort |
| 1 | 300 | 31 | validation cohort |
| 1 | 180 | 39 | validation cohort |
| 1 | 360 | 60 | validation cohort |
| 1 | 240 | 12 | validation cohort |

| 1 | 90 | 46 | validation cohort |
| --- | --- | --- | --- |
| 1 | 270 | 30 | validation cohort |
| 1 | 180 | 149 | validation cohort |
| 0 | 300 | 40 | validation cohort |
| 1 | 300 | 31 | validation cohort |
| 1 | 180 | 39 | validation cohort |
| 1 | 360 | 60 | validation cohort |
| 1 | 240 | 12 | validation cohort |
| 1 | 120 | 67 | validation cohort |
| 1 | 30 | 150 | validation cohort |
| 1 | 90 | 67 | validation cohort |
| 1 | 30 | 30 | validation cohort |
| 1 | 60 | 166 | validation cohort |
| 0 | 540 | 37 | validation cohort |
| 1 | 150 | 37 | validation cohort |
| 1 | 60 | 60 | validation cohort |
| 1 | 30 | 37 | validation cohort |

| 1 | 150 | 37 | validation cohort |
| --- | --- | --- | --- |
| 1 | 60 | 60 | validation cohort |
| 1 | 30 | 37 | validation cohort |
| 1 | 270 | 51 | validation cohort |
| 1 | 150 | 58 | validation cohort |
| 1 | 150 | 39 | validation cohort |
| 1 | 210 | 46 | validation cohort |
| 1 | 120 | 46 | validation cohort |
| 1 | 300 | 46 | validation cohort |
| 1 | 360 | 67 | validation cohort |
| 1 | 90 | 166 | validation cohort |
| 1 | 90 | 166 | validation cohort |
| 0 | 180 | 31 | validation cohort |
| 1 | 270 | 31 | validation cohort |
| 1 | 30 | 78 | validation cohort |
| 1 | 210 | 39 | validation cohort |
| 1 | 150 | 60 | validation cohort |
| 1 | 60 | 67 | validation cohort |

| 0 | 180 | 31 | validation cohort |
| --- | --- | --- | --- |
| 1 | 270 | 31 | validation cohort |
| 1 | 30 | 78 | validation cohort |
| 1 | 210 | 39 | validation cohort |
| 1 | 150 | 60 | validation cohort |
| 1 | 60 | 67 | validation cohort |
| 0 | 180 | 33 | validation cohort |
| 1 | 90 | 40 | validation cohort |
| 1 | 60 | 37 | validation cohort |
| 1 | 180 | 30 | validation cohort |
| 1 | 360 | 30 | validation cohort |
| 1 | 360 | 66 | validation cohort |
| 1 | 90 | 166 | validation cohort |
| 1 | 90 | 46 | validation cohort |
| 1 | 270 | 30 | validation cohort |
| 1 | 180 | 149 | validation cohort |
| 0 | 300 | 40 | validation cohort |
| 1 | 300 | 31 | validation cohort |
| 1 | 180 | 39 | validation cohort |
| 1 | 360 | 60 | validation cohort |
| 1 | 240 | 12 | validation cohort |

| 1 | 120 | 67 | validation cohort |
| --- | --- | --- | --- |
| 1 | 30 | 150 | validation cohort |
| 1 | 90 | 67 | validation cohort |
| 1 | 30 | 30 | validation cohort |
| 1 | 60 | 166 | validation cohort |
| 0 | 540 | 37 | validation cohort |
| 1 | 150 | 37 | validation cohort |
| 1 | 60 | 60 | validation cohort |

| 1 | 90 | 40 | validation cohort |
| --- | --- | --- | --- |
| 1 | 60 | 37 | validation cohort |
| 1 | 180 | 30 | validation cohort |
| 1 | 360 | 30 | validation cohort |
| 1 | 360 | 66 | validation cohort |
| 1 | 90 | 166 | validation cohort |

| 1 | 360 | 67 | validation cohort |
| --- | --- | --- | --- |
| 1 | 90 | 166 | validation cohort |
| 1 | 90 | 166 | validation cohort |
| 0 | 180 | 31 | validation cohort |
| 1 | 270 | 31 | validation cohort |
| 1 | 30 | 78 | validation cohort |
| 1 | 210 | 39 | validation cohort |
| 1 | 150 | 60 | validation cohort |
| 1 | 60 | 67 | validation cohort |
| 0 | 180 | 33 | validation cohort |
| 1 | 90 | 40 | validation cohort |
| 1 | 60 | 37 | validation cohort |
| 1 | 180 | 30 | validation cohort |
| 1 | 360 | 30 | validation cohort |
